# Risk Factors for Hepatocellular Carcinoma: Findings from a Case-Control Study in Vietnam

**DOI:** 10.21203/rs.3.rs-8383243/v1

**Published:** 2026-03-31

**Authors:** Gia Anh Pham, Vu Khanh An Le, Thi Hai Yen Pham, Sy Lanh Nguyen, Lan Huong Nguyen, Chinh Nghia Quach, Manh Thau Cao, Thi Hanh Nguyen, Thanh Xuan Thai, Tuan Anh Luong, Thi Thuy Pham, Duy Hai Vu, Hai Thanh Tran, Thi Lan Le, Hai Vu, Binh Giang Tran, Hung N. Luu

**Affiliations:** Viet Duc University Hospital, Vietnam; Viet Duc University Hospital, Vietnam; UPMC Hillman Cancer Center; Viet Duc University Hospital, Vietnam; Viet Duc University Hospital, Vietnam; Viet Duc University Hospital, Vietnam; Viet Duc University Hospital, Vietnam; Viet Duc University Hospital, Vietnam; Viet Duc University Hospital, Vietnam; Viet Duc University Hospital, Vietnam; Viet Tri University of Industry; Hanoi University of Science and Technology; Hanoi University of Science and Technology; Hanoi University of Science and Technology; Hanoi University of Science and Technology; Viet Duc University Hospital, Vietnam; Houston Methodist Research Institute

**Keywords:** Risk factors, hepatocellular carcinoma, MASLD, smoking, alcohol drinking, Vietnam

## Abstract

**Background.:**

Liver cancer with a major type of hepatocellular carcinoma (HCC) is a leading cancer worldwide and in Vietnam, a low-and -middle income country in the Southeast Asia. While hepatitis B infection is an established risk factor little is known about other non-viral factors for HCC risk. We, therefore, conducted a hospital-based case-control study among 30 HCC patients and 118 healthy controls in Hanoi, Vietnam.

**Methods.:**

HCC was confirmed histopathologically while healthy controls were recruited at the same time in the same locations as HCC cases. Unconditional logistic regression was used to generate odds ratios (ORs) and 95% confidence intervals (CIs) for HCC in relations with potential risk factors.

**Results.:**

Overall, age, sex, diabetes status, platelet, INR, AST, ALT, GGT, albumin, HDL, FIB-4 score, total bilirubin, and AST/ALT ratio were associated with increased risk of HCC. The ORs and respective 95% Cis were 1.08 (1.04–1.12) (per age incremental); 8.72 (3.04–24.95) (male vs. female); 10.05 (1.2–84.03) (diabetes compared with no-diabetes); 32.65 (6.6–161.44) (INR>1.0 compared with ≤1.0); 14.29 (4.52–45.25) (AST^3^40U/L compared with <40U/L); 4.40 (1.73–11.18) (ALT<41 U/L compared with ^3^41U/L); 12.06 (3.9–37.28) (albumin <35g/L compared with 35–50 g/L); 5.27 (1.69–16.47) (HDL≤0.9mmol/L compared with >0.9mmol/L); 4.20 (1.45–12.19) (FIB-4 1.3–2.67 compared with <1.3); 1.06 (1.01–1.10) (per unit incremental-total bilirubin); and 1.62 (1.03–2.26) (per unit incremental-NSF score). Other important factors, including BMI, smoking status, drinking status, cardiometabolic condition, cholesterol, triglyceride, and non-invasive fibrosis score (i.e., AST/ALT score, APRI, BARD) were not.

**Conclusions.:**

Our findings, for the first time, provided evidence of non-viral important risk factors for HCC in Vietnam.

## INTRODUCTION

In 2024, liver cancer was ranked 6^th^ most common cancer and 3^rd^ leading cause of cancer death worldwide, with annually close to 900,000 new cases and approximately 800,000 deaths worldwide.^1^ In Vietnam, primary liver cancer accounts for 15.4% of total new cases and 22.1% of total deaths, making it the leading malignancy in the country.^2^ Similar to other countries, in Vietnam, hepatocellular carcinoma (HCC) is the most common histological subtype *(~90–95% of all primary liver cancer cases).^3^ Established major risk factors for HCC are chronic infection with hepatitis B (HBV) and hepatitis C virus (HCV), excessive alcohol use, and to a lesser extent, dietary exposure to aflatoxin in certain regions.^3^ Given the diminishing role of HBV (due to universal vaccination program) and HCV (available curative therapy), metabolic dysfunction-associated steatotic liver disease (or MASLD),^4^ a nomenclature of non-alcoholic fatty liver disease (or NAFLD) becomes an emerging risk factor for HCC.

Vietnam is a low-middle income country (LMIC), situated in the Southeast Asia area with a total population of 100 million and a significant hepatitis B infection burden.^5^ A recent community-based seroprevalence study of 17,600 adults in Ho Chi Minh City, Vietnam, Pham et al. reported that the prevalence of HBsAg (+) and HBV-naïve were 7.5% and 37.7%, respectively. They also found that the HBV vaccination rates were 18.7% and approximately 50% of population had been exposed to HBV.^5^ While HBV infection is still an important risk factor for HCC development in Vietnam, other risk factors, including tobacco smoking or alcohol drinking are equally important risk factors for HCC as well. A National Health Survey in Vietnam found that 51.2% Vietnamese men were smokers, most of them were exclusive cigarette smokers whereas 23.2% smoked only waterpipe tobacco and about 8% smoked both cigarettes and waterpipe.^6^ Also, a cross-sectional survey among 5,200 participants in 12 provinces in Vietnam showed that nearly three-quarters of males (77%) and one-quarter of females (23%) were current alcohol drinkers.^7^ To our knowledge, only one hospital-based case-control study among 191 HCC cases and 241 controls was conducted in early 90’s during which they reported that alcohol drinking a risk factor for HCC while tobacco smoking was not.^8^ During the past two decades, Vietnam observed rapid development and expansion of economy, coupled with widespread adoption of the western lifestyle and improve life expectancy.^9^ Other potential risk factors for HCC, including cardiometabolic condition, BMI, diabetes as well as the MASLD have not been adequately examined in Vietnam.

To fill this gap of knowledge, we evaluated potential risk factors for HCC in Vietnamese population in a case-control study conducted in Hanoi, Vietnam.

## METHODS

### Study Population

The present analysis was based on data that was derived from hospital-based case–control study including 30 patients with hepatocellular carcinoma (HCC) and 118 healthy controls. Study participants were recruited at the Viet-Duc University Hospital during November 1, 2020 and December 31, 2024 period. All participants provided written informed consent prior to enrollment. The study protocol was approved by the Institutional Review Boards of the participating institutions, including Viet Duc University Hospital and the University of Pittsburgh. The current study was conducted following the Declaration of Helsinki.

### Recruitment of HCC Patients

Patients with hepatocellular carcinoma (HCC) were recruited from the Department of Surgical Oncology during the study period. All patients underwent surgical treatment, and the diagnosis of HCC was confirmed histopathologically by a senior pathologist (Dr. Nguyen L. S.).

### Recruitment of Healthy Control Subjects

Healthy control participants were recruited during the same period that the HCC patients were enrolled from three departments at Viet Duc University Hospital, including the Department of Surgical Oncology, the Department of Diagnostic Imaging, and the Department of General Examination. Eligibility criteria for healthy controls included: (1) age between 40 and 79 years; (2) body mass index (BMI) of 20 to <35 kg/m^2^; (3) no history of metabolic dysfunction–associated steatotic liver disease (MASLD), diabetes, or other conditions specified in the MASLD inclusion/exclusion criteria; and (4) provided written informed consent.

Inclusion criteria for MASLD cases were^4^ 1) Age 40–79 years and 2) non-alcoholic consumption; and 3) steatosis confirmed using the international criteria^10^ [i.e., CAP score ≥ 237 (steatosis) or kPa ≥ 5.5 (fibrosis]; and 3) at least one out of five cardiometabolic criteria (i.e., obesity/overweight, diabetes, hypertension or hyperlipidemia) as follow: [a] BMI >23 kg/m^2^ or waist-circumference >94 cm for male participants or 80cm for female participants; or [b] Diabetes diagnosis (i.e., Fasting serum glucose ^3^5.6 mml./L (100mg/dL) or 2-hour post-load glucose levels ^3^ 7.8mmol/L (^3^140mg/dL) or HbA1c^3^5.7% (39mmol/L) or type 2 diabetes or Treatment for type 2 diabetes); or [c] hypertension or CVD diagnosis (i.e., Blood pressure ^3^135/85mmHg or specific antihypertensive drug treatment); or [d] Plasma triglycerides ^3^1.70 mmol/L (150mg/dL) or Lipid lowering treatment; or [e] Plasma HDL-cholesterol ≤ 1.0mmol/L (40mg/dL) (Male) and ≤ 1.3mmol/L (50mg/dL) (Female) or Lipid lowering treatment. We used the following exclusion criteria to exclude those who were not MASLD patients: (1) alcoholic liver disease or consumption of alcohol >14 drinks/week for women or >21 drinks/week for men in the past 12 months; (2) other chronic liver diseases including autoimmune hepatitis, biliary cirrhosis, hemochromatosis and Wilson’s disease; (3) positive serological test for HBsAg, HBV DNA, antibodies to HCV or HCV RNA; (4) the presence of HCC), (5) history of liver transplantation and/or decompensated cirrhosis; (6) cancer under active treatment; (7) uncontrolled psychiatric syndrome; (8) serious comorbidities (e.g., unstable cardiovascular disease and dialysis); and (9) use of antibiotics for ^3^7 days within the past 60 days.

### Exposure Information Collection

A structured questionnaire was used to collect sociodemographic and exposure-related information from all study participants. During in-person interviews, trained interviewers obtained data on: (1) sociodemographic characteristics; (2) lifetime tobacco and alcohol use; (3) height and weight; (4) dietary habits; (5) family history of cancer; (6) history of medication use; and (7) occupational exposures.

### Liver stiffness measurement (LSM)

We used FibroScan model 430 (EchoSens, Paris, France) to measure the liver stiffness, following instructions and training provided by manufacturer^11^. Briefly, measurements were performed on the right lobe of the liver through intercostal spaces with the patient lying in dorsal decubitus with the right arm in maximal abduction. The tip of the probe transducer was covered with coupling gel and placed on the skin, between the ribs at the level of the right lobe of the liver. The operator, assisted by ultrasound time-motion and A-mode images provided by the system, located a portion of the liver that is at least 6cm thick and free of large vascular structures. Once the area of measurement is located, the operator pressed the probe button to begin an acquisition. The measurement depth will be between 25 and 45mm. Ten successful acquisitions were performed on each patient. The median value represented the liver elastic modulus. Evaluation was only performed on 10 successful acquisitions, in which the reliability was considered at least 60% success rate. The success rate was calculated as the number of successful measurements divided by the total number of measurements. The liver stiffness was expressed in kiloPascal (kPa).

### Non-invasive fibrosis scores

Five non-invasive liver fibrosis scores were calculated in this study. (1) The APRI (Aspartate Aminotransferase to Platelet Ratio Index) was calculated as AST (/upper limit of normal)/platelet count (x 10^9^/L)x100.^12^ (2) The AST/ALT (Aspartate aminotransferase/ Alanine aminotransferase) ratio. (3) The FIB-4 (Fibrosis index based on 4 factors) was calculated as age (year)xAST (U/L)/platelet count (x10^9^/L)x[ALT(U/L)]^1/2^.^13^ The cut-off of FIB-4 for NAFLD patients was be adopted from Caucasian population (i.e., 0.97 for F0–2 and 1.98 for F3–4)^14^ and the FIB-4 was validated in Japanese population (i.e., 1.13 for F0–2 and 3.17 for F3–4)^15^. (4) The nonalcoholic fatty liver disease fibrosis score (NFS) score was calculated as −1.675+0.037xage (years)+0.094xBMI(kg/m^2^)+1.13ximpaired fasting glycemia (IFG)/diabetes (yes=1, no=0)+0.99xAST/ALT ratio-0.013xplatelet (x10^9^/L)- 0.66xalbumin (g/dL).^16^ And (5) the BARD score (or Body mass index (BMI), AST/ALT ratio (AAR), and presence of type 2 diabetes mellitus (DM2)) was the weighted sum of three variables (BMI ≥ 28=1 point, AST/ALT ratio ≥ 0.8=2 points, diabetes=1 point).^17^

### Statistical Analysis

We calculated means and standard deviation (SD) for continuous variables and counts and proportions for categorical variables. Differences in continuous variables between cases and controls were assessed using Student’s *t* test or analysis of variance (ANOVA), as appropriate, and differences in categorical variables were evaluated using the χ^2^ test.

Unconditional logistic regression models were used to examine the association between potential risk factors and HCC risk, with results reported as odds ratios (ORs) and corresponding 95% confidence intervals (CIs). The following factors were included in the multivariable logistic regression models, including 1) age, 2) sex, 3) BMI, 4) smoking status (yes vs. no), 5) alcohol drinking status (yes vs. no), 6) family history of cancer (yes vs. no), 7) diabetes status (yes vs. no), 8) cardiometabolic condition (yes vs. no), 9) platelet (>200 vs. ≤200G/L), 10) international normalized ration (INR) (≤1.0 vs. >1.0), 11) AST (<40 vs. ^3^40U/L), 12) ALT (<41 vs. ^3^41U/L), 13) gamma-glutamyl transferase (GGT or transpeptidase) (<55738 vs. ^3^55738U/L), 14) albumin (35–50, <35 and >50g/L), 15) cholesterol (≤5.2 vs. >5.2mmol/L), 16) triglyceride (<1.7 vs. ^3^1.7mmol/L), 17) high-density lipoprotein (HDL) (>0.9 vs. ≤0.9mmol/L), 18) low-density lipoprotein (LDL) (<4.0 vs. ^3^4.0mmol/L), 19) hemoglobin A1c (HbA1c or glycated hemoglobin) (<5.7% vs. ^3^5.7%), 20) FIB-4 (<1.3 vs. 1-3-2.67 and ^3^2.67), 21) kPA (≤8.2 vs. >8.2), 22) CAP score (≤257.1 vs. >257.1), 23) total bilirubin, 24) NFS score and 25) BARD score.

The SAS version 9.4 (SAS Institute Inc, Cary, NC, USA) was used to perform all statistical analyses. All tests were two-sided and *P*-value <0.05 was considered a threshold for a statistically significant level.

## RESULTS

The current analysis included 30 HCC cases and 119 healthy controls. HCC cases were significantly older than healthy control. The respective mean and SD age at enrolment for HCC cases and controls were 56.9 (12.5) years old versus 41.9 (11.5) years old. The sociodemographic characteristics and selected clinical factors of study participants are presented in Table 1. Compared with healthy controls, HCC patients were more likely to be male, more likely to be alcohol drinkers but less likely to be cigarette smokers, more frequent with infection of hepatitis B, more likely to have diabetes and other comorbidity conditions. They had lower platelet count but higher INR score, higher levels of AST, ALT, and GGT but lower level of albumin, compared to those in healthy controls. Also, compared to healthy controls, HCC patients had lower level of triglyceride but higher level of glucose as well as higher score of FIB-4 but lower score of NFS (all *P*’s<0.05). There were no statistically significant differences between HCC patients and healthy controls regarding to BMI, smoking pack-years, family history of cancer, bilirubin level, CAP score, AST/ALT ratio and BARD score (all *P*’s>0.05).

Table 2 presents results of risk factors for HCC in our analysis. Accordingly, age, sex, diabetes status, platelet, INR, AST, ALT, GGT, albumin, HDL, FIB-4 score, total bilirubin, and AST/ALT ratio were associated with increased risk of HCC. The ORs and respective 95% Cis were 1.08 (1.04–1.12) (per age incremental); 8.72 (3.04–24.95) (male vs. female); 10.05 (1.2–84.03) (diabetes compared with no diabetes); 32.65 (6.6–161.44) (INR>1.0 compared with f-45.25) (AST^3^40U/L compared with <40U/L); 4.40 (1.73–11.18) (ALT<41 U/L compared with ^3^41U/L); 12.06 (3.9–37.28) (albumin <35g/L compared with 35–50 g/L); 5.27 (1.69–16.47) (HDL≤0.9mmol/L compared with >0.9mmol/L); 4.20 (1.45–12.19) (FIB-4 1.3–2.67 compared with <1.3); 1.06 (1.01–1.10) (per unit incremental of total bilirubin); and 1.62 (1.03–2.26) (per unit incremental of NSF score) (Table 2).

## DISCUSSION

In the study of 30 HCC patients and 118 healthy controls in the Vietnamese population, we found that while age, sex, total bilirubin, diabetes status, INR, platelet, AST, ALT, GGT, albumin, HDL, FIB-4, kPA, CAP score, and NFS score were risk factors for HCC, other important factors, such as BMI, smoking status, drinking status, cardiometabolic condition, cholesterol or triglyceride as well as other non-invasive fibrosis score (i.e., AST/ALT score, APRI, BARD) were not.

In a case-control study between 1989 and 1992 among 192 HCC cases and 241 controls, Cordier et al.^8^ found that alcohol drinking was associated with increased risk of HCC (OR=2.0, 95& CI: 1.0–4.0, 1–39 g/day compared with none or occasional) while tobacco smoking was none (OR=0.6, 95% CI: 0.2–1.5 for cigarette smokers only and OR=1.1, 95 CI: 0.4–3.0 for waterpipe smokers only when compared with non-smokers). In our analysis, both smoking and drinking were associated with increased risk of HCC in the univariable regression model, however, such associations were diminished in the multivariable regression model.

Our findings that diabetes was associated with increased risk of HCC in the current study is consistent with results from the meta-analysis, conducted by Wang et al.,^18^ which comprised 17 case-control studies and 32 cohort studies. Accordingly, they reported that diabetes was associated with increased risk of HCC in combined analysis (rate ratio-RR=2.31, 95% CI: 1.87–2.84). The pooled risk estimate (odds ratio) of 17 case-control studies was 2.40 (95% CI: 1.85–3.11) and the rate ratio of 25 cohort studies was 2.23 (95% CI: 1.68–2.96). Also, while prior meta-analysis among 116 datasets, conducted by Esposito et al.,^19^ found that metabolic syndrome was associated with increased risk of liver cancer in men (relative risk-RR=1.43, 95% CI: 1.23–1.65) but not in women (RR=1.40, 95% CI: 0.80–2.62), we did not see this association in our analysis. One of the main discrepancies between our analysis and Esposito et al.^19^ was that we used “cardiometabolic condition” while they used “metabolic syndrome” definitions.

One interesting finding is that platelet was associated with increased risk of HCC in our analysis. The primary function of platelets in hemostasis is to initiate the coagulation cascade following vascular injury. Upon exposure to the extracellular matrix (ECM), platelets adhere to it and become activated by contact with collagen and contribute to the formation of a blood clot.^20^ When activated, platelets release α-granules and dense granules that are rich in inflammatory cytokines, chemokines and different growth factors, including insulin-like growth factor 1 (IGF-1), tumor necrosis factor alpha (TNFα), epidermal growth factor (EGF), interleukin-6 (IL-6), chemokine (C-X-C motif) ligand 4 (CXCL4), vascular endothelial growth factor A (VEGF-A), or fibroblast growth factor (FGF).^21^ Platelets are actively recruited to the liver following injury and play important role in tissue regeneration, primarily by releasing high levels of serotonin and stimulating hepatocyte proliferation.^20^ Platelets also interact with other cell types in the stroma, such as hepatic immune cells, endothelial cells, and hepatic stella cells.^22,23^ Beside on their involvement in pro-fibrinogenic signaling, hepatic immune response and mediating interaction between these factors in the stroma, platelets have been known for promoting HCC cell proliferation and invasion (Review by Pavlovic et al.).^24^

Another interesting finding is that low level of albumin was associated with increased risk of HCC, a finding that was reported in a prior study.^25^ Accordingly, Umerhara et al.^25^ studied 415 patients with chronic hepatitis C after IFN therapy and found that those with albumin level <4 g/dL was associated with increased risk of HCC. The OR and 95% was 0.25 (0.10–0.83) among those with albumin level higher than <4 g/dL compared to individuals with albumin level less than <4 g/dL. Similarly, in a prospective cohort study of 2.932 patients with chronic hepatitis B infection in China, Song et al.^26^ also observed that low level of albumin was associated with increased risk of HCC. The hazard ratios-HRs and respective 95% CIs were 1.71 (0.50–5.91), 4.61 (1.46–14.30), and 9.27 (2.69–31.91), *P*_*trend*_=4.92 × 10^−7^, for individuals with albumin level at ≥ 35.8g/L, ≥ 28.5g/L and <28.5g/L, compared with individuals with albumin level ≥ 41.5g/L.

While age appeared to be an important risk factor for HCC in the current analysis, it is equally interesting to note that male also was associated with increased risk of HCC this population, an observation that is consistent with prior findings in animal model study.^27^ Accordingly, Keng at al.^27^ reported that sexual dimorphism in HCC is entirely abolished in Foxa1- and Foxa2-deficient mice, following diethylnitrosamine-induced liver cancer. In normal male and female mice, Foxa1/2 co-regulate target genes alongside androgen receptor (AR) or estrogen receptor (ERα), respectively, during hepatocarcinogenesis. However, this co-regulation is lost in mice lacking Foxa1/2. Consequently, both estrogen-driven protection and androgen-driven promotion of HCC depend on the presence of Foxa1/2. Estrogen appears to play a role in IL-6 production and modulation of gene expression via Foxa transcription factors.

We also found that FIB-4, one of important non-invasive scores, based on biochemical and clinical markers, for diagnosis of MASLD, was also associated with increased risk of HCC in our analysis. The FIB-4 index has been demonstrated to have a prognostic value for predicting adverse liver-related outcomes in MASLD patients^28^ and adverse outcome in MASLD patients^29–31^ Our finding is consistent with a retrospective cohort study among adult patients with NASH cirrhosis (n=1.338) in the US, in which Albhaisi et al. reported that patients with FIB-4 score >3.25 had 147% increased risk of HCC (HR= 1.47, 95% CI: 0.89–2.45) in comparison with those whose FIB-4 <1.45. Similarly, we also found that higher NFS score was associated with increased risk of HCC, a finding that is consistent with a result from a retrospective study that examined records of close to 26,000 Korean individuals who underwent health checkups between September 2004 and December 2005. Accordingly, Kim et al.^32^ found that a high NFS score indicated an 87% increase in risk of cancer, including HCC.

Our study has several limitations. First, selection bias is inescapable due to the nature of study design, a hospital-based case-control. Second, residual confounding might occur due to the uncontrollable confounding factors in the multivariable regression model. Third, because the population catchment area of this study was in northern Vietnam, our study findings is not generalizable to entire Vietnam or other countries or different populations. Finally, the sample size of HCC patients was modest, thus interpretation of our study findings should be cautious.

The present study also has several strengths. To our knowledge, except another study conducted by Cordier et al.^8^ in early ‘90 of 20^th^ century, this might be the first study in Vietnamese population in the 21^st^ century that determine the potential risk factors for HCC. Second, we could minimize the potential effects of confounding factors because we used a comprehensive set of covariates, including clinical and socioeconomic factors.

In summary, in a hospital-based case-control study in Vietnamese population, we showed that age, sex, total bilirubin, diabetes status, INR, platelet, AST, ALT, GGT, albumin, HDL, FIB-4, kPA, CAP score, and NFS score were risk factors for HCC. Findings from the current study pave the way for the tailored- program in HCC surveillance among Vietnamese population in the current context of occurrence of both viral and non-viral factors for HCC development in Vietnam. Further studies in Vietnamese population with larger sample size and in different geographical locations are warranted to reproduce our findings.

## Figures and Tables

**Table 1.** Characteristics of the Study Participants in the Current Study

	Cases (n=30)	Controls (n=118)	P-value
Age, Mean (SD)	56.9 (12.5)	41.9 (11.5)	**<0.0001**
Sex			
Male	53 (88.3%)	49 (44.5%)	**<0.0001**
Female	7 (11.7%)	61 (55.5%)	
BMI, Mean (SD)	21.6 (2.33)	22.1 (2.39)	0.36
Smoking status			
No	53 (88.3%)	49 (44.5%)	**<0.0001**
Yes	7 (11.7%)	61 (55.5%)	
Smoking pack-years, Mean (SD)	7.2 (4.67)	26.6 (22.88)	0.22
Drinking status			
No	39 (65.0%)	105 (89.0%)	**0.0001**
Yes	21 (35.0%)	13 (11.0%)	
HBV status			
Negative	15 (25.0%)	118 (100.0%)	**<0.0001**
Positive	45 (75.0%)	0 (0.0%)	
Diabetes			
No	45 (75.0%)	115 (97.5%)	**<0.0001**
Yes	15 (25.0%)	3 (2.5%)	
Comorbidity			
No	0 (0.0%)	101 (85.6%)	**<0.0001**
Yes	60 (100.0%)	17 (14.4%)	
Family history of cancer			
No	7 (12.1%)	27 (23.7%)	0.08
Yes	50 (86.2%)	87 (76.3%)	
Unknown	1 (1.7%)	0 (0.0%)	
Platelet	204.6 (92.04)	253.5 (56.02)	**<0.0001**
INR, Mean (SD)	15.2 (58.61)	2.9 (14.04)	**<0.0001**
AST (U/L), Mean (SD)	56.0 (33.12)	23.7 (9.63)	**<0.0001**
ALT (U/L), Mean (SD)	53.0 (34.21)	25.3 (20.44)	**<0.0001**
GGT (U/L), Mean (SD)	93.9 (78.54)	28.9 (25.97)	**<0.0001**
Bilirubin, Mean (SD)	10.2 (8.62)	7.6 (3.57)	0.36
Albumin (g/L), Mean (SD)	37.9 (4.96)	42.6 (3.76)	**<0.0001**
Cholesterol (mmol/L), Mean (SD)	4.3 (1.12)	4.7 (1.10)	**0.007**
Triglyceride (mmol/L), Mean (SD)	1.6 (0.69)	1.7 (1.66)	0.08
HDL (mmol/L), Mean (SD)	1.1 (0.30)	1.3 (0.35)	**<0.0001**
LDL (mmol/L), Mean (SD)	71.4 (499.21)	3.1 (1.73)	**<0.0001**
Glucose (mmol/L), Mean (SD)	6.3 (2.41)	4.8 (0.82)	**<0.0001**
FIB4, Mean (SD)	4.2 (10.57)	0.9 (0.45)	**<0.0001**
kPA, Mean (SD)	21.7 (19.65)	4.1 (1.27)	**<0.0001**
CAP score, Mean (SD)	205.0 (47.00)	207.8 (41.20)	0.55
APRI, Mean (SD)	1.0 (1.07)	0.2 (0.12)	**<0.0001**
AST/ALT ratio, Mean (SD)	1.2 (0.56)	1.2 (0.53)	0.31
NFS score. Mean (SD)	−0.7 (4.70)	−2.9 (1.07)	**<0.0001**
BARD score. Mean (SD)	1.8 (0.92)	1.7 (0.74)	0.45

**Abbreviations:** ALT: Alanine aminotransferase; AST: Aspartate aminotransferase; APRI: Aspartate Aminotransferase to Platelet Ratio Index; BARD: Body mass index (BMI), AST/ALT ratio (AAR), and presence of type 2 diabetes mellitus (DM2); BMI: body mass index; CAP: Controlled attenuation parameter; FIB-4: Fibrosis index based on 4 factors; GGT: Gamma-glutamyl transferase; HDL: High-density lipoprotein; INR: international normalized ratio; kPA: kilopascals; LDL: Low-density Lipoprotein; NFS: nonalcoholic fatty liver disease fibrosis score; SD: standard deviation.

**Table 2.** Risk Factors for Hepatocellular Carcinoma in the Current Analysis

	Cases	Controls	Univariable Model OR (95% CI)	Multivariable Model OR (95% CI)
Sex^a^				
Female	7	69	1.00	1.00
Male	53	49	**4.20 (1.45–12.19)**	**8.72 (3.04–24.95)**
BMI^b^				
≤23 kg/m^2^	41	79	1.00	1.00
>23 kg/m^2^	19	39	0.94 (0.48–1.83)	0.48 (0.20–1.15)
Smoking status^b^				
No	54	115	1.00	1.00
Yes	6	3	**4.26 (1.03–17.68)**	1.26 (0.27–5.88)
Drinking status^b^				
No	39	105	1.00	1.00
Yes	21	13	**4.35 (1.99–9.52)**	1.67 (0.63–4.41)
Family history of cancer^b^				
No	7	27	1.00	1.00
Yes	50	87	2.22 (0.9–5.46)	3.03 (0.97–9.49)
Diabetes^c^				
No	45	115	1.00	1.00
Yes	35	3	**12.78 (3.53–46.26)**	**10.05 (1.2–84.03)**
Cardiometabolic condition^d^				
No	6	25	1.00	1.00
Yes	54	93	2.42 (0.93–6.27)	1.33 (0.37–4.81)
Platelet (G/L)^d^				
>200	98	28	1.00	1.00
≤200	20	32	**5.60 (2.78–11.27)**	**2.95 (1.20–7.26)**
INR^d^				
≤1.0	4	65	1.00	1.00
>1.0	55	46	**19.42 (6.58–57.36)**	**32.65 (6.6–161.44)**
AST (U/L)^d^				
<40	21	112	1.00	1.00
^3^40	39	6	**34.66 (13.04–92.15)**	**14.29 (4.52–45.25)**
ALT (U/L)^d^				
<41	24	102	1.0	1.00
^3^41	36	16	**9.56 (4.57–20.00)**	**4.40 (1.73–11.18)**
GGT (U/L)^d^				
<55738	18	94	1.00	1.00
^3^55738	31	18	**8.99 (4.17–19.41)**	**12.06 (3.9–37.28)**
Albumin (g/L)				
35–50	45	113	1.00	1.00
<35	15	3	12.56 (3.47–45.47)	**5.92 (1.11–31.47)**
>50	0	2		--
Cholesterol (mmol/L)^d^				
≤5.2	42	78	1.00	1.00
>5.2	9	37	0.45 (0.20–1.03)	0.38 (0.13–1.11)
Triglyceride (mmol/L)^d^				
<1.7	33	79	1.00	1.00
^3^1.7	19	36	1.26 (0.63–2.52)	0.66 (0.25–1.71)
HDL (mmol/L)^d^				
>0.9	34	105	1.00	1.00
≤0.9	17	10	**5.25 (2.20–12.55)**	**5.27 (1.69–16.47)**
LDL (mmol/L)^d^				
<4.0	48	100	1.00	1.00
^3^4.0	4	15	0.56 (0.17–1.76)	0.73 (0.16–3.25)
Glucose (mmol/L)^d^				
≤6.1	40	113	1.00	1.00
>6.1	19	5	10.73 (3.76–30.63)	3.25 (0.61–17.24)
HbA1c (%)^d^				
<5.7	9	44	1.00	1.00
^3^5.7	21	15	6.84 (2.58–18.17)	0.60 (0.09–3.94)
FIB4^d^				
<1.3	15	104	1.00	1.00
1.3–2.67	25	14	12.38 (5.30–28.94)	**4.20 (1.45–12.19)**
^3^2.67	20	0	--	--
kPA^d^				
≤8.2	16	117	1.00	1.00
>8.2	43	1	**314.40 (40.47–2442.0)**	**115.97 (13.88–969.2)**
CAP score^d^				
≤257.1	53	110	1.00	1.00
>257.1	6	7	1.78 (0.57–5.56)	1.46 (0.3–7.17)
Age^d^	60	118	**1.11 (1.07–1.15)**	**1.08 (1.04–1.12)**
Total bilirubin^d^	60	118	**1.06 (1.02–1.09)**	**1.06 (1.01–1.10)**
APRI^d^	60	118	--	407.48 (17.33-. )
AST/ALT ratio^d^	60	118	0.83 (0.46–1.51)	0.97 (0.42–2.23)
NFS score^d^	60	118	**2.53 (1.84–3.49)**	**1.52 (1.03–2.26)**
BARD score^d^	60	118	1.09 (0.73–1.62)	0.88 (0.51–1.51)

**Abbreviations:** ALT: Alanine aminotransferase; AST: Aspartate aminotransferase; APRI: Aspartate Aminotransferase to Platelet Ratio Index; BARD: Body mass index (BMI), AST/ALT ratio (AAR), and presence of type 2 diabetes mellitus (DM2); BMI: body mass index; CAP: Controlled attenuation parameter; FIB-4: Fibrosis index based on 4 factors; GGT: Gamma-glutamyl transferase; HDL: Highdensity lipoprotein; INR: international normalized ratio; kPA: kilopascals; LDL: Low-density Lipoprotein; NFS: nonalcoholic fatty liver disease fibrosis score.

a Model adjusted for Age, BMI, diabetes

b Model adjusted for Age, Sex, diabetes

c Model adjusted for Age, Sex, BMI

d Model adjusted for Age, Sex, BMI, diabetes

## Data Availability

The data that support the findings of this study are available from the corresponding author upon reasonable request.
